# Predictors and outcome of surgical repair of obstetric fistula at a regional referral hospital, Mbarara, western Uganda

**DOI:** 10.1186/1471-2490-11-23

**Published:** 2011-12-07

**Authors:** Musa Kayondo, Ssalongo Wasswa, Jerome Kabakyenga, Nozmo Mukiibi, Jude Senkungu, Amy Stenson, Peter Mukasa

**Affiliations:** 1 Mbarara University of Science and Technology, Faculty of Medicine, P.O.BOX 1410, Mbarara, Uganda; 2 Department of Obstetrics and Gynaecology Mbarara Regional Referral Hospital, P.O.BOX 1410, Mbarara, Uganda; 3 Department of Obstetrics and Gynaecology University of California, Los Angeles, USA; 4 EngenderHealth Uganda and Fistula Care Project, P.O.BOX, 34016 Kampala, Uganda

## Abstract

**Background:**

Obstetric fistula although virtually eliminated in high income countries, still remains a prevalent and debilitating condition in many parts of the developing world. It occurs in areas where access to care at childbirth is limited, or of poor quality and where few hospitals offer the necessary corrective surgery.

**Methods:**

This was a prospective observational study where all women who attended Mbarara Regional Referral Hospital in western Uganda with obstetric fistula during the study period were assessed pre-operatively for social demographics, fistula characteristics, classification and outcomes after surgery. Assessment for fistula closure and stress incontinence after surgery was done using a dye test before discharge

**Results:**

Of the 77 women who were recruited in this study, 60 (77.9%) had successful closure of their fistulae. Unsuccessful fistula closure was significantly associated with large fistula size (Odds Ratio 6 95% Confidential interval 1.46-24.63), circumferential fistulae (Odds ratio 9.33 95% Confidential interval 2.23-39.12) and moderate to severe vaginal scarring (Odds ratio 12.24 95% Confidential interval 1.52-98.30). Vaginal scarring was the only factor independently associated with unsuccessful fistula repair (Odds ratio 10 95% confidential interval 1.12-100.57). Residual stress incontinence after successful fistula closure was associated with type IIb fistulae (Odds ratio 5.56 95% Confidential interval 1.34-23.02), circumferential fistulae (Odds ratio 10.5 95% Confidential interval 1.39-79.13) and previous unsuccessful fistula repair (Odds ratio 4.8 95% Confidential interval 1.27-18.11). Independent predictors for residual stress incontinence after successful fistula closure were urethral involvement (Odds Ratio 4.024 95% Confidential interval 2.77-5.83) and previous unsuccessful fistula repair (Odds ratio 38.69 95% Confidential interval 2.13-703.88).

**Conclusions:**

This study demonstrated that large fistula size, circumferential fistulae and marked vaginal scarring are predictors for unsuccessful fistula repair while predictors for residual stress incontinence after successful fistula closure were urethral involvement, circumferential fistulae and previous unsuccessful fistula repair.

## Background

Each year pregnancy related complications claim the lives of over 500,000 women worldwide with about 99% of these deaths occurring in developing countries [1]. Current estimates indicate that for each woman who dies from pregnancy related complications, another 15 to 30 suffer serious morbidities including obstetric fistula, and all these are preventable and treatable conditions [2].

An obstetric fistula is an abnormal connection between the vagina and bladder (vesicovaginal fistula) and/or the rectum (rectovaginal fistula), resulting in uncontrollable leakage of urine and/or stool and often resulting from prolonged obstructed labour. The constant pressure of the fetal presenting part against the soft tissues around the vagina and bladder and or rectum causes ischemic necrosis, leaving a hole behind with leakage occurring after 3-5 days and the extent of injury depends on the duration of labour [3].

The urethrovesical junction is affected in most cases but any other site along the genital tract may be involved like the bladder base, urethra with partial or complete urethral loss and detachment (circumferential fistula), extensive anterior vaginal wall loss or just beside the cervix (juxtacervical fistula). With this prolonged obstruction, the fetus dies in about 95% of cases, the head softens and a stillbirth is delivered if the mother manages to survive [4].

Various classifications of obstetric fistula have been developed but none of these has been adopted internationally. Waaldijk (1995) developed a classification based on the distance of the fistula from the external urethral orifice (EUO) and its effect on the closing mechanism of the urethra classifying Obstetric fistula as type I, II and III; Type I (fistula greater than 5 cm from the external urethral orifice and does not affect the urethral closing mechanism), Type II (fistula less than 5 cm from the external urethral orifice and involves the closing mechanism. Type II is further subdivided into IIa and IIb depending on presence or absence of urethral involvement), Type III fistulae denote any others like ureteric and intracervical fistulae [5]

The leakage of urine and/or stool leaves the women with a persistent odour resulting into serious consequences like isolation from society due to shame, rejection by husbands, loss of status and dignity, and loss of income as they are not able to work for a living [6]. Obstetric fistula is rarely seen in developed countries with good obstetric care yet it continues to cause untold suffering to many women in the developing world [7].

Approximately 2-3.5 million women may be living with fistula worldwide, with 50,000-100,000 new cases occurring annually, most of which are in the Sub-Saharan Africa and Asia [1,2,6,8-10],. The exact prevalence in Uganda is not well known but current national figure stands at 2.6% translating to 200,000 women in reproductive age [11].

In 2001, the Ministry of Health in Uganda, recognized obstetric fistula as a silent morbidity among Ugandan women that evoked interest among policy makers and implementers and one year later, a National Fistula Technical Working Group comprising of donors, non government organisations, local partners and united nations agencies in reproductive health was formed and it generated the process of developing a coherent, definitive national strategy for obstetric fistula elimination [12,13].

Already, obstetric fistula is incorporated in the reproductive health policy that aims at integrating the management of obstetric fistula into existing reproductive health services at all levels of health care, improving access to obstetric fistula management and rehabilitation services and eliminating factors that cause obstetric fistula by increasing awareness about Obstetric fistula and providing appropriate obstetric care [13].

Although the potential for repair of fistula exists at all regional referral hospitals and general hospitals, obstetric fistula repair is routinely done in 17 facilities in the country, the majority of patients getting treatment in Obstetric fistula surgical camps organised by local and visiting fistula surgeons who also mentor up-coming surgeons [14].

Approximately 80% of women with obstetric fistula never seek treatment due to lack of awareness, affordability or access. The cost of repairing a fistula is about $100-$400 which is far beyond what most patients can afford. Even those who are able to seek treatment have to travel long distances to repair centres and wait for weeks to get repaired due to various constraints like a heavy backlog of cases, few surgeons, shortage of supplies and equipment among others [8,15,16].

Information from various literature shows that obstetric fistula appears to be linked to certain social-economic and cultural factors including young age at marriage, poverty and illiteracy, living in rural areas with lack emergence obstetric care [6,7,16-21]

Obstetric fistula has serious social and economic consequences on the lives of these women. The majority of the women are abandoned by their spouses who cannot stand the smell of urine. Divorce rates due to fistula are about 50% in Nigeria while in Zambia it is as low as 15% [6,18].

Various studies have shown that obstetric fistula usually affects first time mothers who have laboured for several days at home, with no access to emergency obstetric care including life saving procedures like caesarean section. These women end up with obstructed labour, stillbirths and for those who survive this ordeal, an obstetric fistula often develops [6,10,18,20,21].

Women with obstetric fistula are usually small and short which is an indication of pelvic immaturity and cephalopelvic disproportion [4,6,9,10,17,22] and most of them develop vesico-vaginal fistula, recto-vaginal fistula or both.

Success rate after repair of obstetric fistula varies from centre to centre and is determined by many factors like site of fistula, degree of scarring, previous repair attempts, repair technique and expertise of the surgeon, equipment and post operative nursing care among others,. Success rates after repair are as high as 87-93% [8,15,16,23,24]. But even after successful closure 15-20% of cases may continue to suffer from urinary incontinence and the predictors of failure include vaginal scarring, circumferential fistula and previous attempts of repair [10,18,25-28]. The aims of this study were to determine 1) the outcome of surgical repair of obstetric fistula 2) the factors that predict outcomes of surgical repair of obstetric fistula at a regional referral hospital, Mbarara, Uganda

## Methods

### Study setting

The study setting was Mbarara University Teaching and Regional Referral Hospital which is located in Mbarara Municipality, which is 286 km south west of Kampala the capital city of Uganda. It is a public hospital funded by the Government of Uganda through Ministry of Health. It is the referral hospital for south western Uganda serving 10 districts with a population of more than 2.5 million people. It also receives patients from neighbouring countries of Rwanda, Tanzania and Democratic Republic of Congo. It is the teaching hospital for Mbarara University of Science and Technology medical school. It handles about 10,000 deliveries per year. There are two fistula surgeons who are competent in repairing 'simple fistula' and therefore are able to do routine fistula repairs, but also organise two fistula surgical camps in a year supported by visiting local and international fistula surgeons

### Study Design

This was a prospective observational study of all women admitted with obstetric fistula from 1^st ^February 2010 to 30^th ^June 2010. Those who presented with a history of obstetric fistula for less than two months were excluded from the study. A total of 77 women with obstetric fistulae were recruited for this study.

### Data collection and procedures

Data was collected on socio-demographic variables: Age, religion, district of residence, marital status, parity, obstetric characteristics: antenatal care attendance, time spent in labour, place of delivery, mode of delivery, outcome of delivery, parity at fistula development, time spent with fistula and previous repairs and physical examination findings (height, weight, type of fistula, fistula classification and size, level of vaginal scarring and outcome of repair at discharge). Outcome of repair was determined using a dye test at discharge 14-21 days later. The outcome of repair was determined on discharge using a dye test.

### Procedures

All patients eligible for the study were admitted on the gynaecology ward and their socio-demographic profile taken. Pre-operatively all women were assessed and the fistula classified using the Waaldijk classification 1995 basing on the size and site of fistula plus level of scarring. The patients were operated using the principles of fistula surgery. All patients with Vesico-vaginal fistula stayed on the ward for 14-21 days with an indwelling urinary catheter depending on the operative findings and were followed up daily during the ward rounds to assess their progress. At discharge, a dye test was done to determine the outcome of repair (successful closure with continence, successful closure with stress incontinence and failed repair). We considered a successful repair with continence if a women was are dry following fistula surgery after 14-21 days of continuous bladder drainage, successful repair with incontinence if a women was wet of urine on stress but had a negative dye test after catheter removal and Failure or Unsuccessful closure if a women was wet of urine and had a positive dye test after 14-21 days of continuous bladder drainage following fistula

### Data analysis

Data was entered into the data base of Epidata and later exported to Statistical Package for Social Scientists (SPSS) version 10 for analysis. Descriptive analysis was performed using means, medians, ranges, standard deviations and proportions. Chi-square test was used to test for association between categorical variables like class of fistula, site of fistula, size of fistula, degree of urethral involvement and level of vaginal scarring with outcome of repair. Mann-Whitney test was used to compare differences in medians for continuous variables that were not normally distributed like participants' age and time spent with fistula with surgical repair outcome. A bivariate regression was used to determine the level of association between the categorical variables and the outcome of repair. Multivariate analysis was used to determine the variables that independently predicted unsuccessful repair and successful repair with residual stress incontinence. Association was considered significant at a p-value less than 0.05

### Ethical considerations

Ethical approval was obtained from Mbarara University Institutional Ethical Research Committee and Informed consent was sought from all participants.

## Results

Most of the fistula patients were primiparous 32 (41.6%), had some formal education 47 (61%) and 35 (45.5%) were still married despite having a fistula. Fifty four (70%) of the participants had antenatal care attendance in the causative pregnancy and 59 (76.6%) delivered from a health facility. Most of the participants had been delivered by caesarean section 46 (59.7%) and the perinatal mortality was as high as 90% [Table 1]

**Table 1:** Participants' Demographic and Obstetric characteristics

Characteristic	Categories	Frequency (N = 77)	Percentage
Age Categories (years)	17 and less	2	2.6
	18 to 34	50	64.9
	35 above	25	32.5
Education level	No formal education	30	39
	Lower primary (P1-P4)	38	49.3
	Upper primary (P5-P7)	9	11.7
Marital status	Single	1	1.3
	Married	35	45.5
	Divorced/separated	36	46.8
	Widowed	5	6.5
Parity at fistula development	Primepara	43	55.8
	Multipara	18	23.4
	Grand multipara	16	20.8
Mode of delivery	Spontaneous vaginal delivery	23	29.9
	Caesarean section	46	59.7
	Instrumental delivery	8	10.4
Any antenatal care attendance in the causative pregnancy		54	70
Delivery from a health unit		59	76.6
Perinatal mortality		70	90

Majority of the participants had been in labour for an average of 2.5 days and the mean age at fistula development was 24 years (standard deviation 7.8).

Among the vesico-vaginal fistula 35 (46%) were of small size, 28 (37.8%) involved the urethra and 32 (41.6%) had been previously repaired unsuccessfully.

Circumferential fistulae accounted for 42.9% in those who had urethral involvement while in 50 (64.9%) of the participants, the level of vaginal scarring was moderate to severe [Table 2].

**Table 2:** Fistula characteristics among study participants

Fistula characteristic	Proportion	Percentage
Fistula class		
Type I	33	45
Type IIa	13	17
Type IIb	28	37
Type III	1	1.3
Level of urethral involvement		
Partial/Juxta urethral	16	57.1
Circumferential	12	42.9
Fistula size [n = 76]		
Small (< 1 cm)	35	46
Medium (1-3 cm)	27	36
Large (> 3 cm)	14	18
Previous repair	32	41.6
Level of vaginal scarring		
Mild/No	27	35.1
Moderate-Severe	50	64.9

Most of the participants with vesico-vaginal fistula had successful repair of their fistulae 55 (79.7%) and 42 (76.2%) were successfully repaired with continence.

**Figure 1:**
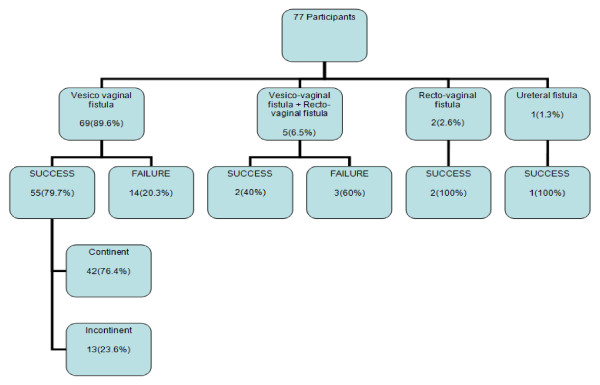
**Fistula types among study participants in relation to outcome of repair**.

A large proportion of those with both vesico-vaginal fistula and recto-vaginal fistula 3 (60%) had unsuccessful closure of the fistulae [Figure 1].

The participants in this study originated from twelve districts. Majority were from Isingiro district 17 (22.1%) while Kiruhura district had the highest proportion of women with unsuccessful fistula repair 5 (62.5%). All clients from Masaka and Bushenyi districts had successful repair of the obstetric fistulae. The median age of participants with successful repair (29 years, interquartile range 22-43.75) was higher than that of those with unsuccessful repair (27 years, interquartile range 23.5-37). However there was no significant difference in the median ages of the two populations (Mann-Whitney = 505.5, p = 0.96).

The median duration with fistula in the group who had successful repair (5.5 years, interquartile range 1-11.75 years) was higher than that of those in which repair was unsuccessful (5 years, interquartile range 1-7.5 years) but this was not significant (p = 0.68).

Fistula size, urethral involvement and level of vaginal scarring were significantly associated with unsuccessful fistula repair [Table 3].

**Table 3:** Fistula characteristics associated with unsuccessful fistula repair among study participants

Variable	Categories	Proportion with unsuccessful repair n (%)	P-value
	Large (> 3 cm)	7 (50)	
Fistula size	Medium (1-3 cm)	5 (18.5)	**0.021**
	Small (< 1 cm)	5 (14.3)	
Previous repair	Previously repaired	8 (25)	0.60
	Not previously repaired	9 (20)	
	Type III	0 (0)	
Fistula class	Type IIb	11 (39.3)	0.06*
	Type IIa	2 (15.4)	
	Type I	4 (12.1)	
	Circumferential	7 (58.5)	
Level of urethral involvement	Partial/Juxta urethral	4 (25)	**0.004**
	No urethral involvement	6 (13)	
Level of vaginal scarring	Moderate-severe	16 (32)	0.004*
	Mild/no scarring	1 (3.7)	

Result interpreted using Fisher's exact test.

Participants with large fistulae were six (6) times more likely to have unsuccessful repair than those with small fistulae. This was statistically significant (Odds ratio 6, 95% Confidential interval 1.46-24.63)[Table 4]

**Table 4:** Fistula characteristics that predicted unsuccessful fistula repair among study participants

Variable	Categories	Unadjusted Odd ratio (CI)
	Large (> 3 cm)	6 (1.46-24.63)**
Fistula size	Medium (1-3 cms)	1.36 (0.35-5.29)
	Small (< 3 cms)	1
Level of vaginal scarring	Moderate to Severe scarring	12.24 (1.52-98.30)*
	Mild or no scarring	1
	Circumferential	9.33 (2.23-39.12)**
Level of Urethral involvement	Partial/Juxta Urethral involvement	2.22 (0.54-9.20)
	No urethral involvement	1
Fistula Classification	Type III	0.15
	Type IIb	4.69 (1.29-17.07)
	Type IIa	1.32 (0.21-8.25)
	Type I	1

**-p < 0.001, *-p < 0.05.

Clients with moderate-severe vaginal scarring were twelve (12) times more likely to have failure of repair than those with mild or no scarring and this was significant (Odds ratio 12.24, Confidential interval 1.52-98.3).

Participants with circumferential fistulae were nine (9) times more likely to fail on repair than cases where the urethra was not involved and this was significant (Odds ratio 9.33 95% Confidential interval 2.23-39.12) [Table 4].

Moderate-severe vaginal scarring was the only characteristic independently associated with unsuccessful fistula repair (Odds ratio 10 95% Confidential interval 1.12-100.57)

Fistula class, urethral involvement, previous unsuccessful fistula repair and level of vaginal scarring were significantly associated with successful fistula repair with residual stress incontinence [Table 5]. Participants with circumferential fistulae were ten (10) times more likely to have residual stress incontinence upon successful closure of their fistulae than those without urethral involvement and this was significant (Odds ratio 10.5, 95% Confidential interval 1.39-79.13)[Table 5].

**Table 5:** Fistula characteristics that predicted successful fistula repair with residual stress incontinence among study participants

Variable	Categories	Unadjusted Odd ratio (CI)
Fistula size	Large (> 3 cm)	5.33(0.93-30.50)
	Medium (1-3 cm)	0.63(1.39-2.86)
	Small (< 3 cm)	1
Level of vaginal scarring	Moderate severe	NA
	Mild or no scarring	1
Urethral involvement	Circumferential	10.50 (1.39-79.13)*
	Partial/Juxta	5 (1.14-22.00)*
	Urethral involvement	
	No urethral involvement	1
Class of Fistula	Type II b	5.56(1.34-23.02)*
	Type II a	O.625(0.06-6.30)
	Type I	1
Previous Repair	Yes	4.8(1.27-18.11)*
	No	1

**-p < 0.001, *-p < 0.05.

Those with type IIb Fistulae were six (6) times more likely to end up with stress incontinence after successful closure than those with type I fistulae. This was significant (Odds ratio 5.56, 95% Confidential interval 1.03-23.02).

Participants with previous unsuccessful repair were five (5) times more likely to end up with residual stress incontinence than those without prior repair and this was significant (Odds ratio 4.8, 95% Confidential interval 1.27-18.11)[Table 5].

Type IIb fistula and previous unsuccessful repair were the only factors independently associated with successful fistula repair with residual stress incontinence (OR 4.024, 95% Confidential interval 2.77-5.83) and (Odds ratio 38.69, 95% Confidential interval 2.13-703.88).

## Discussion

### Socio-demographic characteristics of obstetric fistula clients

Thirty five (45.5%) of the participants in this study were still married and living with their spouses despite having the fistula, and these findings were observed in Zambia [18] where 75.7% of the women were still married. This differs from previous studies [6,10] which seemed to suggest that women with obstetric fistula were neglected by their husbands. This difference in the proportion of women still married is difficult to explain but may be due to differences in culture and religious beliefs in the current study and the Zambian study compared to the other studies.

A high number of participants in this study 47 (61%) had some formal education although 80% had not exceeded primary four. It is not that women with fistula gave up their education to get married since the mean age at first marriage in this study was 18 years. However other studies reported high numbers of women with obstetric fistula with no formal education [10,19,29,30]. This difference may be due to policies like universal primary education which is implemented in Uganda.

### Obstetric characteristics of fistula patients

Majority of participants in this study 43 (55.8%) were primiparous at the time of fistula development which compares well to other studies done in Uganda and Zambia where women with obstetric fistula were primiparous at fistula development [4,18]. This shows that obstetric fistula usually affects first time mothers probably because of pelvic inadequacy leading to cephalopelvic disproportion and risk of obstructed labour

Most women in this study experienced prolonged obstructed labour of up to two and a half days on average and although caesarean section was done in 46 (59.7%) of the participants, there was a high perinatal mortality of 70 (90%). The mean duration of labour was 3 days, 17 (46%) of the participants had been delivered by caesarean section and perinatal mortality in the causative delivery was 90%. Similar findings in Zambia and west Africa [18,21,30] where 62 (54.9%) of the participants spent more than 2 days in labour and although caesarean section was performed in 50% of the cases, the perinatal mortality was still high at 78.1%. The profile is similar probably because all the studies were done in low resource countries where there are numerous delays in accessing emergency obstetric care.

Fifty four (70%) of participants in this study had an antenatal care visit in the pregnancy leading to the fistula, similar to the findings in Zambia and West Africa [18,21,30].

Although over 90% of pregnant women attend antenatal care at least once in Uganda, only 42% deliver in health facilities, there is need to use this opportunity to counsel pregnant mothers and about the importance of birth plan and skilled attendance at birth. The national met need for Emergence obstetric care is low at 40% and the Caesarean Section rate at 2.7% below the recommended minimum of 5% [11]. The national road map for reduction of maternal and new born mortality and morbidity which addresses all the 3 delays focusing on the community, transport and referral, quick and quality skilled care in health facilities has been put in place to prevent and manage obstetric complications [13].

### Fistula characteristics

Majority of clients in the current study 69 (89.6%) had vesico-vaginal fistula and only 2 (2.6%) had recto-vaginal fistula. This compares to previous studies [4,18] where most of the participants had vesico-vaginal fistula. This high frequency of vesico-vaginal fistula compared to other fistulae is probably due to the increased likelihood of anterior vaginal wall compression by the fetal head against the bony pelvis hence causing more ischemia to the bladder than the rectum.

The mean duration with fistula in this study was 7.3 years similar to earlier studies done in Uganda [4]. Since both studies were done in the same country, the obstacles to repair may be the same which could explain the delay in repair. However a study done in Ethiopia [27] found that the mean duration with fistula was 4.8 years among their participants. This difference could be due to the fact that Ethiopia has 2 hospitals (Addis Ababa fistula hospital and Bahirdar fistula centre) that are fully committed to fistula repair hence little backlog of cases.

### Fistula repair outcomes

In this study, successful closure of the genitourinary fistula was achieved in 77.9% of the women. In a study done in Zambia [18], the successful closure rate was 72.9%. The success rates are almost similar probably because the patients' fistula characteristics in both studies may have been similar. However this success rate was lower than that reported by Hancock 2004 which was 90% [4]. This could be due to the difference in fistulae handled in both studies as there was a higher proportion of patients with complex fistulae in the current study (circumferential 12%, moderate to severe scarring 64.9%) compared to those in Hancock's study (circumferential 10%, moderate to severe scarring 3.7%).

Residual stress incontinence in women with successful closure was high (21.7%). A similar proportion was seen in the studies in Ethiopia [22,27]. These results suggest that even after successful fistula closure, stress incontinence is a significant problem. This is because the ischemic process that causes the fistula may also lead to destruction of the urinary continence mechanisms hence leaving the women incontinent even after having the fistulae closed.

### Predictors for unsuccessful fistula repair

In this study, unsuccessful fistula repair was significantly associated with fistula size, urethral involvement and level of vaginal scarring.

Women with large fistulae were six (6) times more likely to have an unsuccessful repair than those with small fistulae (p < 0.01, Odds ratio 6, 95% Confidential interval 1.46-24.63). This is probably due to the fact that large fistulae are difficult to mobilize fully or there is little bladder tissue left to achieve a tension free repair. However in a study to assess the risk of failure of repair of genito-urinary fistula in Ethiopia [27], fistula size was not associated with failure. This could probably be due to the smaller proportions of failure (2.7%) in the Ethiopian study than in this study (22.1%).

Those with circumferential fistulae were nine (9) times more likely to have unsuccessful repair (p < 0.01, Odds ratio 9.33, 95% Confidential interval 2.23-39.12). A similar picture has been observed in other studies [27,28] where women with circumferential fistulae were more likely to have failure of repair of their fistula than those without urethral involvement. This association could be due to the fact that in a circumferential the urethra is usually fixed to the pubic bone and hence difficult to mobilize. It could also be due to the difficulty in anastomosing a detached urethra which is usually shortened by the fistula.

Women with moderate-severe vaginal scarring were twelve (12) times more likely to have unsuccessful fistula closure than those with mild or no scarring and there was a statistical significance, similar associations were observed in other studies [27,28]. This is probably because scar tissue has a poor blood supply hence less likely to heal. Also scarred fistulae are difficult to mobilize from the surrounding tissues like vagina and pubic bone making a tension free repair nearly impossible.

In this study, it was only moderate to severe vaginal scarring that was independently associated with unsuccessful fistula repair (p < 0.05 Odds ratio 10 95% Confidential interval 1.12-100.57). This may be due to the fact that both large and circumferential fistulae are usually extensively scarred to the surrounding tissues as a result of severe ischemic process leading to their formation.

### Predictors for successful fistula closure with residual stress incontinence

Fistula class, level of urethral involvement, previous unsuccessful fistula repair and level of vaginal scarring were significantly associated with residual stress incontinence after successful fistula closure in this study.

Participants with type IIb fistulae (fistula involving the urethra) were six (6) times more likely to have residual stress incontinence after successful closure of their fistulae than those with Type I fistulae (fistula located > 5 cm from external urethral orifice) and there was a significant association (Odds ratio 5.56, 95% Confidential interval 1.34-23.02). A similar picture was seen in other studies [27-29]. This relationship is probably because the urine continence mechanisms are within the urethra rather than urethrovesical junction and these are destroyed by the ischemic process during obstructed labour. Even after repair of the fistula, what is left behind is a urethra with no physiological function.

Women with circumferential urethral fistulae were ten (10) times more likely to have successful fistula closure with residual stress incontinence than those where the fistula did not involve the urethra (Odds ratio 10.5, 95% Confidential interval 1.39-79.13), similar to other studies [27-29] This is probably because circumferential urethral injuries involve extensive tissue loss leading to urethral detachment from the bladder. A scarred non functional urethra is left behind leading to incontinence.

In this study, participants who had had previous unsuccessful fistula repair were five (5) times more likely to remain with residual stress incontinence after successful fistula closure than those who had had no previous repair (Odds ratio 4.8, 95% Confidential interval 1.27-18.11), similar findings have been reported [27-29]. Therefore previous repair is a risk factor for stress incontinence probably because with every repair, a scar is left behind around the urethra which holds it open thus removing any physiological function.

There was no significant association between size of fistula and successful fistula repair with residual stress incontinence. However other studies have found significant relationship between fistula size and outcome of surgery [27-29] It could be expected that the extensive dissection and suturing required in closing the large fistulae, leaves tension and scarring on the urethra which holds it open causing incontinence. This was not the case in the current study probably because of the smaller proportion of large fistulae in this study.

### Study limitations

The study population was small (77 cases), however, we are taking this study as preliminary data and we plan a longer follow up of about 5 years and see if the results will be similar or comparable. Also there could have been challenges in patient selection and assessment, skill and competence of some surgeons, and availability of recommended sutures and equipment and the quality of post operative care.

### Implications for practice

In this study, the success rates are below the 2006 WHO requirement for a fistula centre of more than 85% closure and less than 10% incontinence, this could have been due to the fact that there was a reasonable number of clients with previous repairs, challenges in patient selection and assessment, required skill and competence to handle complicated cases by some of the surgeons and availability of recommended sutures and equipment and the quality of post operative care. Hence there is need to consider all these factors when preparing for fistula surgery so as to improve on the surgical outcomes, having in mind that always the primary surgical attempt carries the highest chance of success.

## Conclusions

Obstetric fistula was more common in primiparous patients, especially those coming from the rural areas. The success rate after fistula repair was as high as 77.9% while the level of stress incontinence after successful fistula closure was also high at 21.9%. The risk of unsuccessful fistula repair was related to parameters such as large fistulae (> 3 cm), circumferential fistulae and marked vaginal scarring (moderate to severe scarring). Successful closure with stress incontinence was significantly more likely to occur in women with type IIb fistulae (fistula involving the urethra), circumferential fistulae, marked vaginal scarring and those with previous unsuccessful fistula repair.

## Competing interests

The authors declare that they have no competing interests.

## Authors' contributions

MK, PKM, WS, AS conceived and designed the study, developed data collection instruments and supervised data collection. JS, NM, JK participated in the testing and finalization of the data collection instruments and coordinated study progress JS, KM and PKM performed the statistical analysis, PKM and KM wrote all versions of the manuscript. All authors read and approved the final manuscript.

## Pre-publication history

The pre-publication history for this paper can be accessed here:

http://www.biomedcentral.com/1471-2490/11/23/prepub
